# Individual characteristics associated with the magnitude of heat acclimation adaptations

**DOI:** 10.1007/s00421-021-04626-3

**Published:** 2021-03-01

**Authors:** Puck Alkemade, Nicola Gerrett, Thijs M. H. Eijsvogels, Hein A. M. Daanen

**Affiliations:** 1 Faculty of Behavioural and Movement Sciences, Vrije Universiteit Amsterdam, Amsterdam Movement Sciences, 1081 HV Amsterdam, The Netherlands; 2 Department of Physiology, Radboud Institute for Health Sciences, Radboud University Medical Center, Nijmegen, The Netherlands

**Keywords:** Inter-individual variation, Controlled hyperthermia, Heat acclimation, Morphology, Maximum oxygen uptake, Physical fitness

## Abstract

**Purpose:**

The magnitude of heat acclimation (HA) adaptations varies largely among individuals, but it remains unclear what factors influence this variability. This study compared individual characteristics related to fitness status and body dimensions of low-, medium-, and high responders to HA.

**Methods:**

Twenty-four participants (9 female, 15 male; maximum oxygen uptake [$\dot{{V}}$O_2peak,kg_] 52 ± 9 mL kg^−1^ min^−1^) completed 10 daily controlled-hyperthermia HA sessions. Adaptations were evaluated by heat stress tests (HST; 35 min cycling 1.5 W  kg^−1^; 33 °C, 65% relative humidity) pre- and post-HA. Low-, medium-, and high responder groups were determined based on tertiles (*n* = 8) of individual adaptations for resting rectal temperature (*T*_re_), exercise-induced *T*_re_ rise (Δ*T*_re_), whole-body sweat rate (WBSR), and heart rate (HR).

**Results:**

Body dimensions (*p* > 0.3) and $\dot{{V}}$O_2peak,kg_ (*p* > 0.052) did not differentiate low-, medium-, and high responders for resting *T*_re_ or Δ*T*_re_. High WBSR responders had a larger body mass and lower body surface area-to-mass ratio than low responders (83.0 ± 9.3 vs 67.5 ± 7.3 kg; 249 ± 12 vs 274 ± 15 cm^2^ kg^−1^, respectively; *p* < 0.005). Conversely, high HR responders had a smaller body mass than low responders (69.2 ± 6.8 vs 83.4 ± 9.4 kg; *p* = 0.02). $\dot{{V}}$O_2peak,kg_ did not differ among levels of responsiveness for WBSR and HR (*p* > 0.3).

**Conclusion:**

Individual body dimensions influenced the magnitude of sudomotor and cardiovascular adaptive responses, but did not differentiate *T*_re_ adaptations to HA. The influence of $\dot{{V}}$O_2peak,kg_ on the magnitude of adaptations was limited.

**Supplementary Information:**

The online version contains supplementary material available at 10.1007/s00421-021-04626-3.

## Introduction

Heat acclimation (HA), i.e., repeated exposures to heat stress within a certain time frame, can be adopted to artificially induce improvements in sweat and skin blood flow responses, fluid balance, cardiovascular stability, and thermal tolerance. These adaptive responses result in a lower thermal strain during exercise at a given workload, which is usually reflected by a lower core temperature, heart rate (HR) and skin temperature, higher whole-body sweat rate (WBSR), and improved thermal comfort (Periard et al. 2015; Daanen et al. 2018).

The magnitude of HA adaptations depends on the intensity, duration, frequency, and number of heat exposures (Taylor and Cotter 2006; Periard et al. 2015). Furthermore, characteristics specific to the individual undertaking HA likely influence the development of adaptations; some authors have suggested the existence of low- and high responders to HA (Taylor and Cotter 2006; Racinais et al. 2012). Identification of low- and high responders to HA is of significant practical importance in occupational, athletic, or military settings, when practitioners or institutions desire to individualize strategies that reduce thermal strain. However, there is limited evidence-based knowledge on what factors may predispose individuals to either group. There is potentially a genetic component that mediates HA responsiveness (Bouchard et al. 2011; Taylor 2014), but phenotypic characteristics such as fitness status and body dimensions may also relate to inter-individual differences in the adaptive response to HA (Pandolf et al. 1977; Taylor 2014; Casadio et al. 2017; Corbett et al. 2018).

Fitness status, typically characterized as maximum oxygen uptake adjusted for body mass ($\dot{{V}}$O_2peak,kg_), has often been considered to affect HA responsiveness, ever since the findings of Pandolf et al. (1977). They observed that the most fit soldiers required only four HA days to achieve complete adaptation, as defined by a rectal temperature (*T*_re_) adaptation plateau, while the least fit participants required 8 days. These findings suggest that well-trained individuals adapt more rapidly during HA, but the authors did not specify the magnitude of adaptation that was achieved by the individual participants. Thus, whether the absolute adaptive gains varied with baseline fitness status remains unknown.

Taylor and Cotter (2006) proposed that endurance-trained individuals have a relatively low adaptive response to HA. That is, the repeated exposure to high core temperatures and sweating inherent to prolonged physical exercise training confers partial heat adaptation (Avellini et al. 1982; Periard et al. 2015; Lamarche et al. 2018a). This exercise-induced partial heat adaptation may reduce the potential for further adaptation. To date, a few studies have examined the influence of baseline fitness status on the magnitude of the HA response. Early observations from Shvartz et al. (1977) supported the hypothesis that well-trained individuals have a reduced scope for HA adaptation. However, this outcome may be specific to their experimental design. The fixed absolute workload during their HA sessions induced greater *T*_re_ rises in untrained participants than in trained participants, which may have resulted in a higher adaptive stimulus for the untrained group. Inter-individual variation in *T*_re_ during HA can be reduced by implementing a controlled-hyperthermia HA protocol, in which core temperature is elevated to and maintained at a pre-determined value during each session. When this technique was adopted, no association was observed between $\dot{{V}}$O_2peak,kg_ and magnitude of adaptations after 10 days of HA (Corbett et al. 2018). This suggests that baseline fitness status does not differentiate the magnitude of the adaptive response to HA, which is in contrast to the theoretical concept proposed by Taylor and Cotter (2006). It should be noted that $\dot{{V}}$O_2peak,kg_ does not directly relate to the exercise-induced partial heat adaptation status. Researchers have proposed that habitual training activity might be more reflective of this partial adaptation status (Lamarche et al. 2018a; Ravanelli et al. 2020). Altogether, there is no consensus on the degree to which fit and unfit individuals benefit from HA.

Previous studies investigating inter-individual variation in the magnitude of HA adaptations have focused primarily on fitness status (Shvartz et al. 1977; Corbett et al. 2018), whilst the role of body characteristics such as body mass, body surface area (BSA), or its ratio (i.e., BSA-to-mass ratio) has not been assessed. In particular, BSA-to-mass ratio is considered to be an important covariant in individual thermoregulation, with heat exchange between body and environment being dependent upon BSA and body heat storage upon body mass (Havenith et al. 1998; Notley et al. 2016). Whether a high or low BSA-to-mass ratio provides an advantage during exercise heat stress depends on the mode of exercise, prescribed exercise intensity, and the environmental conditions (Havenith 2001; Cramer and Jay 2016; Notley et al. 2016). Notley et al. (2016) observed that during light and moderate exercise with matched heat loss requirements, individuals with a high BSA-to-mass ratio are predisposed to dissipate heat via cutaneous vasodilation, while those with a low BSA-to-mass ratio are more dependent on sweat evaporation for heat loss. The authors suggested that individuals have naturally adapted towards the heat loss mechanism that best suits their body size. In line with this, Taylor (2014) hypothesized that, during HA, individuals may predominantly develop their anthropometric-dependent “preferred” heat loss pathway. We speculate that the “preferred” thermoeffector, determined by one’s body dimensions, is more active during HA, resulting in a greater adaptation for that specific thermoeffector. For example, sweat gland activity during HA may be particularly high for individuals with a low BSA-to-mass ratio, resulting in a pronounced sweat gland adaptation. Although it is not known whether a dose–response relationship exists, Buono et al. (2009) did show that sweat gland activity during HA is essential to improve sweating capacity. Alternatively, one could argue that the less-developed thermoeffector has more potential for adaptation and therefore shows high responsiveness. Thus, it remains unknown how, if at all, one’s body dimensions influence the adaptive response to HA.

To summarize, there is little empirical evidence to support theoretical perspectives on the individual adaptive response to HA. Therefore, the objective of our explorative study was to compare individual characteristics related to fitness status and body dimensions of low-, medium-, and high responders to controlled-hyperthermia HA. Considering the limited research available on the factors that contribute to inter-individual variance during HA, we aimed to explore the potential contributors by including participants with a range of characteristics, rather than isolating one prospective influential characteristic. It was recently shown that most phenotypic adaptations within the individual were not interrelated (Corbett et al. 2018). That is, one phenotypic adaptation (e.g., WBSR) can develop independently of others (e.g., HR or resting *T*_re_). In the present explorative study, participants were therefore classified into the respective responder groups for separate adaptation phenotypes; resting *T*_re_, exercise-induced rise in *T*_re_ (Δ*T*_re_), WBSR and HR.

## Methods

### Participants

We recruited 24 healthy volunteers (15 male, 9 female; Table 1). Participants did not reside in a warm environment (> 25 °C air temperature) for longer than 7 days within the 3 months prior to the study. They did not smoke, had no history of heat-related illnesses or cardiovascular complications, and did not have any known issues with thermoregulation. Three participants were taking medication: 70 mg alendronic acid weekly, 500 mg calci-chew, and 7.5 mg mirtazapine daily (control of benign bone tumor); Ritalin (ADHD); methotrexate and folic acid (rheumatoid arthritis). Six females used the combined pill, one used a hormonal intrauterine device, and two reported regular natural menstrual cycles (25–35 days). Participants were asked to consistently maintain habitual medication and supplement intake over the course of the study. As a potential indicator of partial HA status, weekly exercise training time (min) was determined from the self-reported habitual training program (considering the 2 months preceding the study). With the aim of removing sessions that do not evoke thermal strain, swimming sessions were excluded (Avellini et al. 1982). Procedures were approved by the Ethics Committee of the Faculty of Behavioural and Movement Sciences of the Vrije Universiteit Amsterdam (VCWE-2018-160R1), conform the standards set out by the Declaration of Helsinki. Prior to the study, participants were informed about the procedures and provided verbal and written consent.

**Table 1:** Participants’ baseline characteristics

Characteristic	Mean ± SD	Range
Age (yr)	31 ± 8	21–46
Height (cm)	182.4 ± 8.6	168.0–200.0
Body mass (kg)	75.6 ± 10.5	59.3–97.6
BSA (m^2^)	1.97 ± 0.17	1.67–2.29
BSA-to-mass ratio (cm^2^ kg^−1^)	262 ± 16	227–295
Body fat (%)	20.5 ± 5.8	12.9–31.9
$\dot{{V}}$O_2peak,kg_ (mL kg^−1^ min^−1^)	51.9 ± 8.8	36.9–68.6
Weekly exercise time^×^ (min)	341 ± 122	150–690

*SD *standard deviation,* BSA *body surface area, $\dot{{V}}$*O*_*2peak,kg*_ maximum oxygen uptake relative to body mass

^×^Swimming exercise excluded*, n* = 23

### Study design

During the first visit to the laboratory, participants completed a graded exercise test in temperate conditions to determine baseline $\dot{{V}}$O_2peak,kg_ and were, after a short break, familiarized with the heat stress test (HST). On a separate occasion, body dimensions and composition were assessed. Approximately 7 days after the participants first reported to the laboratory, they completed the first HST (HST1). The next day, participants commenced a 10-consecutive-day controlled-hyperthermia HA program. To evaluate adaptive responses, participants performed a second HST (HST2), which was scheduled 48 h after the last HA session. All HST and HA sessions were administered in an environmental chamber (b-Cat B.V., Tiel, The Netherlands), with air temperature 33 °C, relative humidity 65% and minimal air flow. During HA, participants did not engage in any additional exercise training (few exceptions for occasional short light-intensity exercise bouts in temperate conditions). The study was conducted during winter time (Netherlands; Jan–Apr) to minimize acclimatization status.

### Experimental sessions

#### Body dimensions and composition

BSA was calculated from height (stadiometer; Seca 217, Seca, Hamburg, Germany) and body mass (Platform scale, SATEX 34 SA-1 250, Weegtechniek Holland B.V., Zeewolde, The Netherlands), according to the formula proposed by DuBois and DuBois (1916). BSA-to-mass ratio was calculated as BSA divided by body mass. Body fat percentage was assessed using a whole-body dual-energy X-ray absorptiometry scan (Discovery A, Hologic, Inc., Marlborough, MA, USA).

#### Graded exercise test ($\dot{{V}}$O_2peak,kg_)

To determine maximum oxygen uptake, participants completed a graded exercise test on an electrically braked cycle ergometer (Excalibur Sport, Lode B.V., Groningen, The Netherlands) in temperate conditions (22 °C, 32% relative humidity). Cycling started at a power output of 25 W, after which intensity increased with 25 W min^−1^ until volitional exhaustion. During exercise, strong verbal encouragement was given. The rate of oxygen consumption was monitored breath-by-breath using a metabolic cart (Quark CPET, COSMED, Rome, Italy). Values were discarded if they were higher than two standard deviations from the mean within a local 12-s window. The maximum oxygen uptake was defined as the highest 15-s moving average. Maximum oxygen uptake was divided by body mass (i.e., $\dot{{V}}$O_2peak,kg_) and used as an indicator of individual fitness level.

#### Heat stress test

Testing took place throughout the day, but each participant completed their own two HSTs at the same time of day. They were instructed to refrain from caffeine and alcohol consumption, to avoid strenuous exercise, and to report and replicate food and beverage intake during the 24 h preceding the HSTs. To encourage euhydration, participants were asked to drink 500 mL water the evening before and 10 mL kg body mass^−1^ of water during the 3 h prior to the HST. Upon arrival at the laboratory, participants provided a urine sample, from which urine-specific gravity was measured using a handheld refractometer (PAL-10S, Atago Co. Ltd, Tokyo, Japan). A urine-specific gravity value ≤ 1.025 was considered as an indication of sufficient hydration for adults engaging in daily exercise (Kenefick and Cheuvront 2012). Two participants had, each on one occasion, a urine-specific gravity value above 1.025; after consuming 5 mL kg^−1^ of water, they were allowed to resume the experiments. Upon entering the environmental chamber, participants first rested in a chair for 10 min, while stable baseline measures were obtained. They then mounted an electrically braked cycle ergometer (Excalibur Sport, Lode B.V., Groningen, The Netherlands) and commenced cycling at a power output of 1.5 W kg body mass^−1^ for 35 min. This was followed by a 5-min resting period, during which participants consumed a standardized volume of water (3 mL kg body mass^−1^). Next, participants performed a graded exercise test (GXT_HST_), starting at a power output of 1.5 W kg body mass^−1^ with subsequent increments of 25 W min^−1^ until volitional exhaustion. No feedback or encouragement was given during the GXT_HST_. WBSR was calculated as the difference between pre- and post-session nude body mass, corrected for exposure time (g h^−1^; Platform scale, SATEX 34 SA-1 250, Weegtechniek Holland B.V., Zeewolde, The Netherlands). With every weighing procedure, two measurements were performed from which body mass was determined as the average value over a stable 5-s assessment. If the difference between the two measurements was > 0.05 kg, a third measurement was performed.

#### Controlled-hyperthermia HA sessions

Every participant performed 10 controlled-hyperthermia HA sessions at approximately the same time of day (at least within ± 3 h of HST time). Prior to each session, a urine sample was collected to monitor hydration status over the course of HA. The controlled-hyperthermia protocol served to increase *T*_re_ to 38.5 °C in approximately 35 min (referred to as “thermal drive”) and subsequently maintain T_re_ slightly above 38.5 °C for 60 min (referred to as “thermal maintenance”). During thermal drive, participants cycled at a power output that was expected to cause an increase in *T*_re_ to 38.5 °C within the set time window (Excalibur Sport, Lode B.V., Groningen, The Netherlands; or Wattbike Pro, Wattbike B.V., Duivendrecht, The Netherlands). For the first HA session, power output during this phase was determined based on observations during familiarization and HST1. For the subsequent HA sessions, power output was based on observations during the previous HA sessions. Thermal maintenance was achieved by adjusting power output and introducing resting periods when necessary. Participants were allowed to drink ad libitum during all HA sessions. WBSR was calculated as the difference between pre- and post-session nude body mass plus drinking volume, corrected for exposure time.

### General measurements and calculations

During all HST and HA sessions, HR, *T*_re_, and mean skin temperature (*T*_sk_) were monitored continuously. HR was measured at the chest (Polar Vantage-M, Kempele, Finland). *T*_re_ was used as an indicator for body core temperature and assessed using a rectal thermometer (MSR, Seuzach, Switzerland; or Yellow Springs Instruments, Yellow Springs, OH, USA), which was self-inserted 10 cm past the anal sphincter. Local skin temperature was measured at the chest, forearm, thigh, and calf using iButtons (DS1922, Maxim Integrated Products, Inc., San Jose, CA, USA), which were attached to the skin with tape (Fixomull Stretch ADH, BSN Medical GmbH, Hamburg, Germany). *T*_sk_ was calculated as a weighted average of the four local skin temperatures (Ramanathan 1964). To quantify the individual adaptive stimulus during HA, we calculated the approximate integral (trapezoidal numerical integration) of, respectively, the *T*_re_ and *T*_sk_ curves (1-min averages) for each session, and summated the approximated session integrals:

$${\text{Cumulative adaptation impulse X}} = \mathop \sum \limits_{{i_{{{\text{HA}}}} = 1}}^{{N_{{{\text{HA}}}} }} \left( {\mathop \sum \limits_{i = 1}^{N} \frac{{X_{i - 1} + X_{i} }}{2} \cdot \Delta t} \right)_{{i{\text{HA}}}}$$

where *X* = physiological variable (i.e., *T*_re_ or *T*_sk_ [°C·min]); *i*_HA_ = the *i*th HA session (starting from 1); *N*_HA_ = the total number of HA sessions (i.e., 10); *N* = the total number of time intervals (i.e., session duration in minutes); *X*_*i*_ = *X* at the end of the *i*th interval; Δ*t* = time interval in min (i.e., 1).

This approach was adapted from Taylor and Cotter (2006; Taylor 2014), who introduced the “cumulative adaption impulse” as the summated session integrals for mean body temperature. Total work done (J) during HA was calculated using the same equation, where *X* = power output (W) and Δ*t* is expressed in s.

### Data analysis

All data were synchronized and formatted using MATLAB (R2019a, The MathWorks Inc., Natick, MA, USA). Statistical analysis was performed using R software (version 3.6.3, R Foundation for Statistical Computing, Vienna, Austria) in the Rstudio environment (version 1.2.5033, Rstudio, Inc., Boston, MA, USA). Data were reported as mean ± standard deviation. The level of statistical significance was set at *p* ≤ 0.050. When non-parametric tests were performed (as outlined below), data were reported as median (first quartile, third quartile) and *p *values were reported with superscript “np”.

The overall group responses to HA (all participants) were assessed by comparing physiological measures for HST1 and HST2 using paired *t* tests. These HST values were determined as follows: resting *T*_re_ = average *T*_re_ during min 5–10 of baseline rest; end-exercise *T*_re_, HR and *T*_sk_ = average value over the last 5 min of the fixed workload exercise; Δ*T*_re_ = end-exercise *T*_re_ minus resting *T*_re_; WBSR = WBSR over full HST (fixed workload exercise + GXT_HST_); and GXT_HST_ time = time to exhaustion on graded exercise test in the heat. Normality of the HST2–HST1 differences was analyzed using the Shapiro–Wilk test. If the normality assumption was violated (Shapiro–Wilk test yielded *p* ≤ 0.05), the Wilcoxon Signed-Rank test was used to evaluate the overall group responses to HA. To investigate the inter-dependency of physiological adaptations, associations between adaptations were assessed using Pearson’s product–moment correlation coefficient *r*_p_ or Spearman’s rank-order correlation coefficient *r*_s_ (when non-normal or non-linear). The magnitude of adaptations was quantified by subtracting the value at HST1 from the value at HST2 (HST2–HST1). In addition, associations between individual characteristics were assessed to confirm independency. Linearity was confirmed visually and normality of the variables was evaluated using the Shapiro–Wilk test. The strength of the association was classified as trivial (*r* < 0.1), small (*r* = 0.1–0.3), moderate (*r* = 0.3–0.5), or large (*r* > 0.5) (Cohen 1988).

For each adaptation phenotype, participants were divided into one of three equally sized groups (i.e., tertiles); (1) low-, (2) medium- or (3) high responders. We performed multiple one-way analyses of variance (i.e., ANOVAs) to evaluate the null hypothesis that the low-, medium-, and high responders have similar individual characteristics and HST1 responses and received comparable adaptive stimulus during HA. Normality of the residuals was analyzed using the Shapiro–Wilk test. Homogeneity of variance was visually inspected by plotting the residuals against the fitted values. If the residuals were not normally distributed or when residuals showed considerable variance across the range of fitted values, the non-parametric Kruskal–Wallis test was performed to compare the three groups. When a significant effect was observed, post hoc pairwise comparisons were done using independent *t* tests (when assumptions were met) or Wilcoxon Rank Sum tests (when assumptions were not met) with Bonferroni correction. In addition, an alternative analysis using simple least-squares regression is reported in the electronic supplementary material (text and tables in supplementary file1, figures in supplementary file2).

## Results

### HA

Twenty-four participants completed all experimental trials. On average, the target *T*_re_ of 38.5 °C during HA was achieved in 37.5 ± 6.7 min. On 175 occasions, the target *T*_re_ was reached within 40 min, while on 65 occasions, this took between 41 and 61 min. The full HA session duration was 96.5 ± 6.9 min. The average *T*_re_ during the thermal drive and thermal maintenance phase was 37.9 ± 0.2 °C and 38.6 ± 0.09 °C, respectively. HA was performed with a power output of 91 ± 18 W, HR of 132 ± 14 bpm, and WBSR of 1244 ± 444 g h^−1^.

### Physiological responses to HA and their interactions

During HST2, both resting *T*_re_ and end-exercise *T*_re_ were lower than during HST1 (HST1 37.5 ± 0.3 vs. HST2 37.3 ± 0.4 °C, *p* = 0.02; HST1 38.2 ± 0.3 vs. HST2 38.1 ± 0.4 °C, *p* = 0.005; respectively). Δ*T*_re_ remained unchanged (HST1 0.7 ± 0.3 vs. HST2 0.8 ± 0.3 °C, *p* = 0.6). Post-HA, we observed lower values for end-exercise *T*_sk_ (HST1 36.5 ± 0.3 vs. HST2 36.2 ± 0.4 °C, *p* = 0.001) and end-exercise HR (HST1 149 ± 17 vs. HST2 139 ± 15 bpm, *p* < 0.001). WBSR increased following HA (HST1 1017 [787, 1326] vs. HST2 1237 [888, 1756] g h^−1^, *p* < 0.001^np^). Performance at the GXT_HST_ was improved, with a longer time to exhaustion in HST2 (578 ± 132 s) than in HST1 (525 ± 138 s, *p* < 0.001). The resting *T*_re_ adaptation was related to the Δ*T*_re_ adaptation (*r*_p_ = − 0.72, *p* < 0.001) and end-exercise *T*_re_ adaptation (*r*_p_ = 0.51, *p* = 0.01). There was an association between the WBSR adaptation and end-exercise *T*_sk_ adaptation (*r*_s_ = − 0.53, *p* = 0.02). No significant associations were observed among any other adaptation indices.

### Individual characteristics

Weekly exercise training time was related to both body mass (*r* = 0.52, *p* = 0.01) and BSA–mass ratio (*r* = − 0.63, *p* = 0.001). Body fat percentage and $\dot{{V}}$O_2peak,kg_ were significantly correlated (*r* = − 0.79, *p* =  < 0.001). Inherent to their calculation, body mass, BSA, and BSA–mass ratio were interrelated (*r* = − 0.91, *r* = − 0.76, *r* = 0.95, *p* < 0.001).

### Resting *T*_re_ responders

**Fig. 1:**
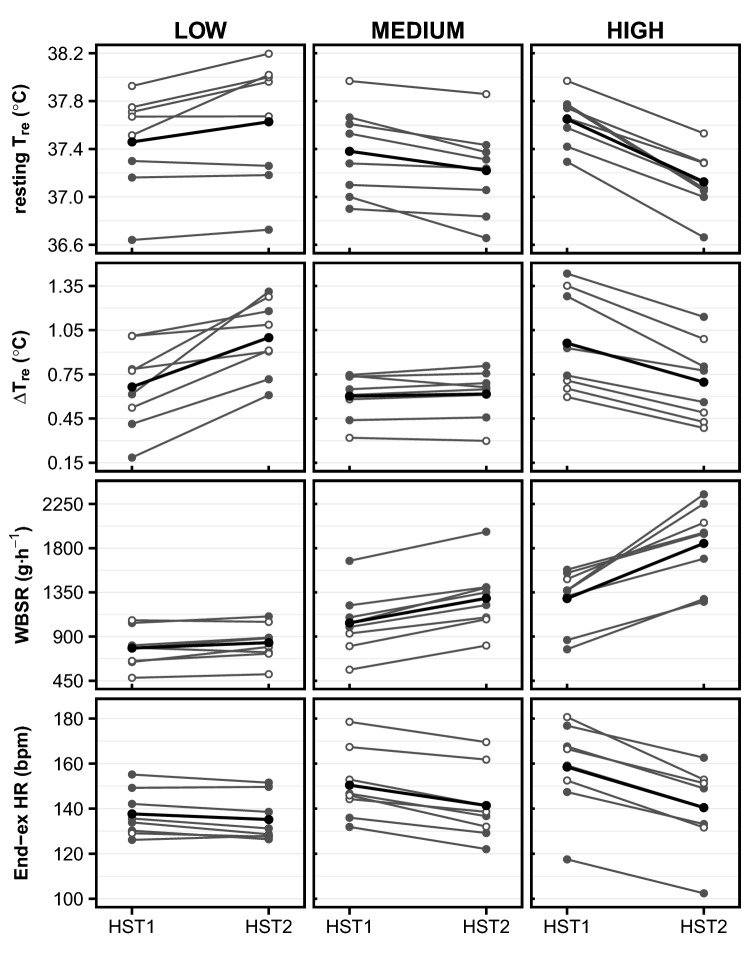
Physiological responses to HSTs in low-, medium-, and high responders for resting *T*_re_ (first row), Δ*T*_re_ (second row), WBSR (third row), and HR (fourth row). *T*_re_, rectal temperature; Δ*T*_re_, exercise-induced rise in rectal temperature; WBSR, whole-body sweat rate; End-ex, end-exercise (average over last 5 min of exercise); HR, heart rate; HST, heat stress test. Gray lines represent individual responses, with filled points showing males and open points showing females. Black lines and points represent the average group response

**Fig. 2:**
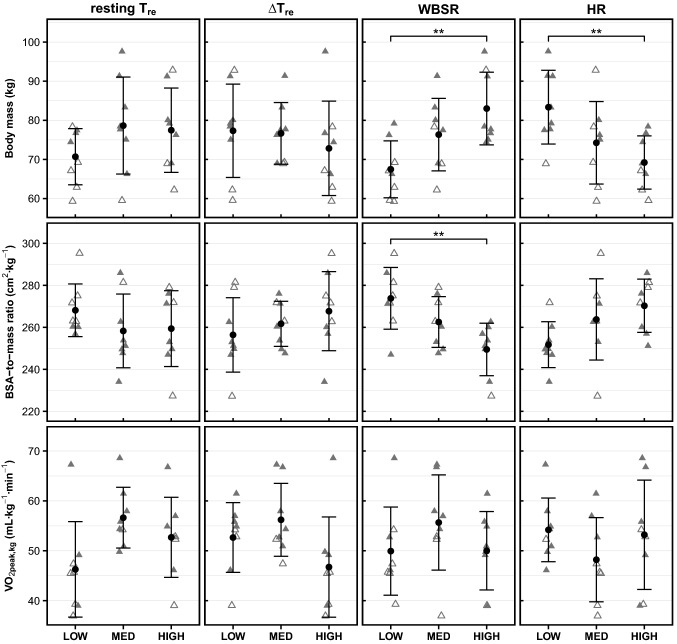
Individual characteristics of low-, medium-, and high responders for resting *T*_re_ (first column), Δ*T*_re_ (second column), WBSR (third column), and HR (fourth column). *T*_re_, rectal temperature; Δ*T*_re_, exercise-induced rise in rectal temperature; WBSR, whole-body sweat rate; HR, heart rate; BSA-to-mass ratio, body surface area-to-mass ratio; $\dot{{V}}$O_2peak,kg_, maximum oxygen uptake relative to body mass; Filled triangles represent males, open triangles represent females. Group means and standard deviations are presented in black. Significance denotations: *p ≤ 0.050, **p ≤ 0.010

**Fig. 3:**
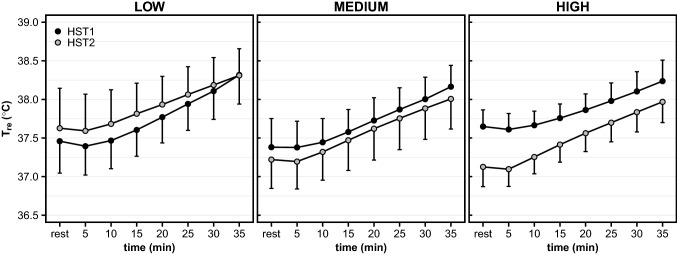
*T*_re_ response during HST1 (black points) and HST2 (gray points) for low-, medium-, and high resting *T*_re_ responders. *T*_re_, rectal temperature; HST, heat stress test. Data are presented with mean and standard deviation at 5-min intervals

As per our grouping criteria, resting *T*_re_ increased or remained unchanged in low resting *T*_re_ responders, while resting *T*_re_ decreased in all high responders following HA (Fig. 1). Body mass and BSA-to-mass ratio were not significantly different between low-, medium-, and high resting *T*_re_ responders (Fig. 2). $\dot{{V}}$O_2peak,kg_ did not vary significantly among resting *T*_re_ responders, but approached statistical significance (*p* = 0.052; Fig. 2). Body fat percentage varied among responder groups, with the highest values for low responders, but no pairwise differences were identified post hoc (*p* > 0.07; Table 2). Considering the inter-dependency of the resting *T*_re_ adaptation and Δ*T*_re_ adaptation, we have displayed the *T*_re_ responses of low-, medium-, and high resting *T*_re_ responders to HST1 and HST2 in Fig. 3.

**Table 2:** Individual characteristics of low-, medium-, and high responders for resting *T*_re_, Δ*T*_re_, WBSR and HR [mean ± SD or median (*Q*1, *Q*3)] with *p *values from analysis of variance

	Resting *T*_re_ responders				Δ*T*_re_ responders			
	Low	Medium	High	*p*	Low	Medium	High	***p***
Pre- to post-HA change^§^ (°C)	0.2 (0.0, 0.3)^m,h^	− 0.1 (− 0.2, − 0.1)^l,h^	− 0.5 (− 0.6, − 0.4)^l,m^	** < 0.001**^**np**^	0.3 (0.2, 0.4)^m,h^	0.0 (0.0, 0.0)^l,h^	− 0.2 (− 0.3, − 0.2)^l,m^	** < 0.001**^**np**^
Sex (no. male/female)	3/5	7/1	5/3	–	5/3	6/2	4/4	–
Age (y)	27 (24, 35)	32 (30, 36)	28 (26, 36)	0.6^np^	34 ± 7	27 ± 5	32 ± 10	0.2
Body mass (kg)	70.7 ± 7.2	78.7 ± 12.4	77.5 ± 10.8	0.3	77.3 ± 11.9	76.7 ± 7.9	72.8 ± 12.1	0.7
BSA (m^2^)	1.9 ± 0.1	2.0 ± 0.2	2.0 ± 0.2	0.3	2.0 ± 0.2	2.0 ± 0.1	1.9 ± 0.2	0.7
BSA-to-mass ratio (cm^2^ kg^−1^)	268 ± 13	258 ± 18	259 ± 18	0.4	256 ± 18	262 ± 11	268 ± 19	0.4
Body fat (%)	25 ± 7	18 ± 4	19 ± 4	**0.04**	19 ± 5	18 ± 5	24 ± 7	0.06
$\dot{{V}}$O_2peak,kg_ (mL kg^−1^ min^−1^)	46.3 ± 9.6	56.6 ± 6.1	52.7 ± 8.0	0.052	52.7 ± 7.0	56.2 ± 7.3	46.7 ± 10.0	0.09
Weekly exercise time^×^ (min)	292 ± 117	385 ± 54	340 ± 166	0.4	406 ± 131	335 ± 115	275 ± 89	0.1

	WBSR responders				HR responders			
	Low	Medium	High	*p*	Low	Medium	High	*p*
Pre- to post-HA change^§^ (g h^−1^/bpm)	70 (23, 84)^m,h^	250 (214, 279)^l,h^	455 (387, 652)^l,m^	** < 0.001**^**np**^	− 4 (− 4, − 1)^m,h^	− 9 (− 10, − 7)^l,h^	− 17 (− 19, − 15)^l,m^	** < 0.001**^**np**^
Sex (no. male/female)	3/5	5/3	7/1	–	7/1	3/5	5/3	–
Age (y)	30 ± 7	30 ± 8	34 ± 9	0.4	31 ± 8	32 ± 8	31 ± 8	0.9
Body mass (kg)	67.5 ± 7.3^h^	76.3 ± 9.3	83.0 ± 9.3^l^	**0.006**	83.4 ± 9.4^h^	74.2 ± 10.5	69.2 ± 6.8^l^	**0.02**
BSA (m^2^)	1.8 ± 0.1^h^	2.0 ± 0.2	2.1 ± 0.1^l^	**0.02**	2.1 ± 0.2^h^	1.9 ± 0.2	1.9 ± 0.1^l^	**0.02**
BSA-to-mass ratio (cm^2^ kg^−1^)	274 ± 15^h^	263 ± 12	249 ± 12^l^	**0.005**	252 ± 11	264 ± 19	270 ± 13	0.06
Body fat (%)	23 ± 6	19 ± 6	19 ± 4	0.3	18 ± 3	23 ± 7	20 ± 6	0.2
$\dot{{V}}$O_2peak,kg_ (mL kg^−1^ min^−1^)	49.9 ± 8.8	55.7 ± 9.5	50.0 ± 7.9	0.3	54.2 ± 6.4	48.2 ± 8.4	53.2 ± 11.0	0.4
Weekly exercise time^×^ (min)	303 ± 95	332 ± 107	385 ± 154	0.4	375 ± 64	353 ± 163	290 ± 118	0.4

*p* values ≤ 0.050 are highlighted in bold

*SD *standard deviation,* Q1 *first quartile,* Q3 *third quartile,* BSA* body surface area, $\dot{{V}}$*˙O*_*2peak,kg*_ maximum oxygen uptake relative to body mass,* T*_*re*_ rectal temperature,* ΔT*_*re*_ exercise-induced rise in rectal temperature,* HR *heart rate,* WBSR* whole-body sweat rate

^np^From non-parametric test

^l^Significantly different from low responders (*p* ≤ 0.050)

^m^Significantly different from medium responders (*p* ≤ 0.050)

^h^Significantly different from high responders (*p* ≤ 0.050)

^§^Pre- to post-HA change was calculated as heat stress test 2 minus heat stress test 1

^×^swimming exercise excluded, *n* = 23

### Δ*T*_re_ responders

As per our grouping criteria, Δ*T*_re_ increased in all low Δ*T*_re_ responders, while Δ*T*_re_ decreased in all high responders following HA (Fig. 1). Body mass, BSA-to-mass ratio, and $\dot{{V}}$O_2peak,kg_ were not significantly different between low-, medium-, and high Δ*T*_re_ responders (Fig. 2). There were significant effects of Δ*T*_re_ responder group on the Δ*T*_re_ and WBSR_BSA_ during HST1 (Table 3). Post hoc pairwise comparisons revealed that individuals with a large Δ*T*_re_ (i.e., within-HST rise in *T*_re_) adaptation had a greater Δ*T*_re_ during HST1 than individuals with a medium Δ*T*_re_ adaptation (high 1.0 ± 0.3 °C vs. medium 0.6 ± 0.2 °C, *p* = 0.047). High Δ*T*_re_ responders had a lower WBSR_BSA_ during HST1 than low Δ*T*_re_ responders (high 411 ± 126 g h^−1^ m^−2^ vs. low 598 ± 117 g h^−1^ m^−2^, *p* = 0.03.

**Table 3:** *p *values for the main effects of responder groups on physiological variables during HST1 and HA. Physiological variables were compared between low-, medium-, and high responders for resting *T*_re_, Δ*T*_re_, WBSR, and HR. p values ≤ 0.050 are highlighted in bold. Details are presented in  the electronic supplementary material (file1), Tables 4–7

	Resting *T*_re_	Δ*T*_re_	WBSR	HR
Physiological responses HST1				
Δ*T*_re_ (°C)	0.3^np^	0.04*	0.2^np^	0.1^np^
End-exercise *T*_re_ (°C)	0.6	0.3	0.7	0.2
End-exercise *T*_sk_ (°C)	> 0.9^np^	0.4	0.7^np^	0.2
WBSR (g h^−1^)	**0.02**^**np**^	0.053	0.007*	0.09
WBSR_BSA_ (g h^−1^ m^−2^)	**0.03**^**np**^	0.02*	0.03*	0.2
HR (bpm)	**0.04**	0.5	0.5	0.048*
Heat acclimation				
Average duration thermal drive phase (min)	0.4	0.7	**0.04**^**np**^	0.2^np^
Cumulative adaptation impulse *T*_re_ (°C·min)	0.5	0.8	**0.02**^np^*	0.3^np^
Cumulative adaptation impulse *T*_sk_ (°C·min)	0.5	0.6^np^	**0.03**^**np**^	0.3^np^
Average HR (bpm)	0.053	0.4	0.9	0.2
Total work done (kJ)	0.4	0.4	0.2	0.06
Average power output (W)	0.5	0.3	0.3	0.1
Average power output thermal drive phase (W)	0.4	0.3	0.2	0.054
Average power output (W kg^−1^)	> 0.9^np^	0.5	0.07	0.3
WBSR (g h^−1^)	**0.04**^**np**^	0.1	0.006*	0.02*
WBSR_BSA_ (g h^−1^ m^−2^)	**0.04**^**np**^	0.1	0.03*	0.03*

*HST* heat stress test,* T*_*re*_ rectal temperature,* ΔT*_*re*_ exercise-induced rise in rectal temperature,* T*_*sk*_ mean skin temperature,* HR* heart rate,* End-exercise *average over last 5 min of exercise,* WBSR *whole-body sweat rate,* BSA* body surface area

^np^From non-parametric test

*Significant pairwise differences were identified in post hoc tests, details provided in text

### WBSR responders

As per our grouping criteria, WBSR increased in all responder groups, with the largest elevation in the high WBSR responders following HA (Fig. 1). High WBSR responders had a significantly larger body mass (*p* = 0.005), BSA (*p* = 0.02), and smaller BSA-to-mass ratio (*p* = 0.004) than low WBSR responders (Fig. 2; Table 2). $\dot{{V}}$O_2peak,kg_ did not vary significantly among WBSR responder groups (*p* = 0.3, Fig. 2). There were significant effects of WBSR responder group on WBSR and WBSR_BSA_ during HST1, the cumulative adaptation impulse for *T*_re_, and the WBSR during HA and the end-exercise *T*_sk_ adaptation (Table 3). Post hoc pairwise comparisons revealed that high WBSR responders had a greater WBSR and WBSR_BSA_ during HST1 than low responders (high 1287 ± 305 g h^−1^ vs. low 782 ± 198 g h^−1^, *p* = 0.005; high 621 ± 126 g h^−1^ m^−2^ vs. low 425 ± 108 g h^−1^ m^−2^, *p* = 0.02). The cumulative adaptation impulse for *T*_re_ was higher in high WBSR responders (37,983 [37211, 38925] °C·min) than low WBSR responders (34,976 [34919, 35926] °C·min, *p* = 0.04^np^). High WBSR responders sweat more during HA than low WBSR responders, as shown by a larger average WBSR (high 1524 ± 297 g h^−1^ vs. low 916 ± 239 g h^−1^, *p* = 0.005) and WBSR_BSA_ (high 738 ± 129 g h^−1^ m^−2^ vs. low 499 ± 137 g h^−1^ m^−2^, *p* = 0.03) during HA.

### HR responders

As per our grouping criteria, end-exercise HR decreased in all responder groups, with the largest reduction in the high HR responders following HA (Fig. 1). High HR responders had a significantly smaller body mass (*p* = 0.02) and BSA (*p* = 0.02) than low responders (Fig. 2; Table 2). $\dot{{V}}$O_2peak,kg_ did not vary significantly among HR responder groups (*p* = 0.4, Fig. 2). There were significant effects of HR responder group on HR during HST1 and WBSR during HA (Table 3). Post hoc pairwise comparisons revealed that high HR responders had a higher HR during HST1 than low responders (high 158 ± 20 bpm vs. low 138 ± 10 bpm, *p* = 0.047). Low HR responders sweat more during HA than medium HR responders, as shown by a larger average WBSR (low 1515 ± 400 g h^−1^ m^−2^ vs. medium 969 ± 342 g h^−1^ m^−2^, *p* = 0.02) and WBSR_BSA_ (low 724 ± 185 g h^−1^ m^−2^ vs. medium 494 ± 154 g h^−1^ m^−2^, *p* = 0.04) during HA.

## Discussion

The objective of the current study was to compare individual characteristics related to fitness status and body dimensions of low-, medium-, and high responders to a 10-day controlled-hyperthermia HA protocol. The respective responder groups were determined for separate adaptation phenotypes; resting *T*_re_, Δ*T*_re_, WBSR, and HR. Our findings suggest that high WBSR responders generally had a large body mass and BSA and low BSA-to-mass ratio, whereas high HR responders typically had a small body mass and BSA. Individuals with a medium or high resting *T*_re_ adaptation tended to be more fit than individuals with a low resting *T*_re_ adaptation, with a higher $\dot{{V}}$O_2peak,kg_ and lower body fat percentage, but no statistically significant differences were observed. To our knowledge, this is the first experimental study that explores the role of body dimensions as an influential factor in HA responsiveness.

### Physiological responses to HA and their interactions

Overall, our HA protocol successfully induced hallmark adaptations associated with HA; a reduced resting *T*_re_, end-exercise *T*_re_, HR, and *T*_sk_ and an elevated WBSR for exercise at a given workload (1.5 W kg body mass^−1^). The average reductions in resting *T*_re_ (− 0.17 °C) and end-exercise HR (− 10 bpm) were similar to previous studies that have adopted a comparable controlled-hyperthermia HA regimen (Patterson et al. 2004; Gibson et al. 2015). The WBSR adaptation following controlled-hyperthermia HA protocols varies, potentially caused by its dependency on ambient temperature during HA (Tyler et al. 2016). We observed no reduction in the exercise-induced *T*_re_ rise (i.e., Δ*T*_re_) following HA; the reduction in end-exercise *T*_re_ (− 0.14 °C) was simply the result of the resting *T*_re_ adaptation. The explanation for this unaltered exercise-induced *T*_re_ rise may be twofold. First, a humid environment, as used in the current study, may provide limited potential for enhanced evaporative cooling (Buono et al. 1998; Patterson et al. 2004). Indeed, we did not observe a statistically significant association between the Δ*T*_re_ adaptation and WBSR adaptation, while Corbett et al. (2018) reported a moderate positive relationship between these variables following a more hot and dry HA (40 °C, 50%RH). We did find a more pronounced end-exercise *T*_sk_ adaptation along with a larger WBSR adaptation, suggesting that sweating cooled the skin. However, the small reduction in end-exercise *T*_sk_ (on average ~ 0.3 °C) was apparently not sufficient to reduce exercising *T*_re_. Second, we observed that the Δ*T*_re_ adaptation and resting *T*_re_ adaptation were inversely related; when individuals had a considerable resting *T*_re_ adaptation, they showed no or even a “negative” Δ*T*_re_ adaptation (Fig. 3). From Newton’s Law of Cooling, it follows that the rate of temperature change of an object is related to the temperature gradient between that object and the environment (here 33 °C; Taylor 2014). The reduced starting temperature of the “object” (i.e., human) after HA may have diminished the potential for dry heat loss in HST2. The latter suggestion rests on the assumption that, before and after HA, sweating was initiated following a fixed change in core temperature rather than at an absolute core temperature threshold (Patterson et al. 2004; Tyler et al. 2016). Altogether, the magnitude of adaptations in the present study are comparable to previous reports. In accordance with Corbett et al. (2018), we show limited inter-dependency between adaptation phenotypes; high or low responsiveness to HA is likely phenotype-specific.

### WBSR responders, HR responders, and body dimensions

We observed that high WBSR responders had a larger body mass and BSA and a lower BSA-to-mass ratio than those with a low sudomotor adaptation (i.e., low responders). Fitness status and body fat percentage did not differentiate low-, medium-, and high WBSR responders. High WBSR responders had a greater WBSR during HST1 than low responders. The latter is in line with Notley et al. (2016), who revealed that, in compensable conditions, large individuals (low BSA-to-mass ratio) were more reliant upon heat loss via the sudomotor pathway (i.e., sweating) than the vasomotor pathway. Our observation that these heavy sweaters develop superior sweat adaptation supports the notion by Taylor (2014) that individuals may adapt towards their “preferred” pathway for heat loss. Taylor (2014) approached this from an evolutionary perspective, but it is also plausible that increased activation of a thermoeffector during HA, by virtue of one’s body dimensions, results in a more pronounced adaptation of that thermoeffector. Since Buono et al. (2009) observed that sweat gland activity during HA is essential to develop sudomotor adaptations, one might suggest that a dose–response relationship exists; the more sweat gland activity during HA, the larger the sweat gland adaptation. Indeed, we found that high WBSR responders lost more sweat in total and per unit of BSA during HA than low responders. This might imply that the high WBSR responders, characterized by a low BSA-to-mass ratio, had a relatively large sweat gland output capacity or active sweat gland density during HA, which enabled superior sweat gland adaptation.

In addition, high WBSR responders showed an elevated cumulative adaptation impulse for *T*_re_ with respect to the low responders. The most conceivable explanation for this is a longer duration of the thermal drive phase during HA sessions; the time to reach a *T*_re_ of 38.5 °C tended to be longer (7 min per HA session) for high WBSR responders. This observation is likely related to the large thermal inertia inherent to their body mass. Since a *T*_re_ of at least 38.5 °C has been recommended to induce complete heat adaptation (Fox et al. 1963; Gibson et al. 2015; Racinais et al. 2015), we believe that this extended thermal drive phase in high WBSR responders did not notably enlarge the adaptive sudomotor stimulus.

In contrast to WBSR, high HR responders were mostly small individuals, with smaller body mass and BSA than low responders. Fitness status and body fat percentage did not differentiate low-, medium-, and high HR responders. HR reductions following HA occur as a result of a lower thermal strain (lower *T*_re_) and/or an expanded plasma volume (Taylor 2014; Tyler et al. 2016; Periard et al. 2016). Among our HR responder groups, *T*_re_ adaptations did not differ, and therefore, it could be speculated that high HR responders had a greater plasma volume expansion than low responders. The HA-induced plasma volume expansion improves cardiovascular stability and increases the specific heat of the blood, with the latter supporting heat transfer from the core to the skin (Periard et al. 2016). This improved heat transfer potentially lowered the cutaneous blood flow demands (Sawka et al. 2011; Periard et al. 2016), allowing cardiovascular strain to decrease substantially during exercise. Thus, the large HR adaptation for small individuals may relate to a superior HA-induced plasma volume expansion, though we cannot confirm this in the present study.

Furthermore, high HR responders had a higher exercising HR during HST1 than low HR responders. In line with this, Corbett et al. (2018) observed that a higher end-exercise HR prior to HA was associated with a larger subsequent HR adaptation. The higher pre-HA cardiovascular strain for high HR responders, most of whom were small individuals (i.e., small body mass) in our study, might be a result of their elevated reliance on the vasomotor pathway to dissipate heat (Notley et al. 2016). That is, during exercise heat stress, HR rises in response to the concurrent blood flow demands of the cutaneous circulation and working skeletal muscle (Sawka et al. 2011; Periard et al. 2016). Increased reliance on dry heat loss might therefore impose higher cardiovascular strain upon small individuals.

The contrasting body dimensions of high WBSR and high HR responders suggest a morphological dependency of the adaptive response to HA. One’s “preferred” heat loss avenue, which is related to one’s body dimensions (Notley et al. 2016), may dictate the adaptive pathway during HA (Taylor 2014). This notion is supported by our observation that heavy sweaters developed superior sweat adaptation. This notion also implies that small individuals, who mainly rely upon dry heat exchange, would develop a more pronounced vasomotor adaptation. The pronounced HR adaptation in small individuals may relate to this hypothesis, but in the present study, we did not implement the appropriate measures to directly confirm this. These inferences may only apply to HA in a warm humid environment, which allows both dry and wet heat exchange (present study; 33 °C, 65% relative humidity). Distinct outcomes may be observed when one of these heat loss pathways is restricted by high ambient temperatures (i.e., exceeding *T*_sk_) or a higher humidity. Data from Notley (2016; dissertation) indicated that HA adaptations were similar for small (273 cm^2^ kg^−1^) and large (244 cm^2^ kg^−1^) individuals in a more hot-dry environment. Future studies should investigate the isolated effect of body dimensions on sudomotor and vasomotor adaptations following HA in various environmental conditions.

### Resting *T*_re_ and fitness status

Medium and high resting *T*_re_ responders tended to be more fit than low responders, with a higher $\dot{{V}}$O_2peak,kg_ and lower body fat percentage, but the evidence was not sufficiently strong to reject the null hypothesis. Body dimensions did not differentiate low-, medium-, and high resting *T*_re_ responders. Our findings contradict the notion that well-trained individuals have a reduced scope for adaptation (Taylor and Cotter 2006). Support for this notion comes from earlier work by Shvartz et al. (1977), who showed greater adaptation in untrained individuals (~ 36 mL kg^−1^ min^−1^) for exercising HR, resting *T*_re_, and exercising *T*_re_ than in trained individuals (~ 60 mL kg^−1^ min^−1^). In that study, however, the untrained individuals had a higher *T*_re_ during HA sessions, resulting in a higher adaptive stimulus during HA, which complicates interpretation of their findings. Our controlled-hyperthermia HA reduced potential bias resulting from inter-individual variation in cumulative adaptation impulse. Indeed, in accordance with our results, Corbett et al. (2018) recently reported that $\dot{{V}}$O_2peak,kg_ (range 45–75 mL kg^−1^ min^−1^) was not associated with the increase in WBSR or the reduction in end-exercise HR, end-exercise *T*_re_ and Δ*T*_re_ after 10 controlled-hyperthermia HA days. Thus, in recreationally active and well-trained participants, fitness status may not affect the magnitude of adaptation following 10 days of controlled-hyperthermia HA.

The notion that $\dot{{V}}$O_2peak,kg_ would affect HA responsiveness rests on the assumption that $\dot{{V}}$O_2peak,kg_ represents partial adaptation status (Taylor and Cotter 2006; Ravanelli et al. 2020). However, this assumption may lack validity, given the variability in $\dot{{V}}$O_2peak,kg_ trainability (Bouchard et al. 2011) and exercise environment (e.g., water vs. land; Avellini et al. 1982) or modality (e.g., sprinters vs. endurance athletes; Amano et al. 2013). We therefore included the self-reported weekly exercise training time (swimming excluded) into our analysis. However, our results must be interpreted with caution considering the response bias in self-reported data. Also, we did not implement a validated physical activity questionnaire (e.g., as in Lamarche et al. 2018b), which might be a more sensitive measure of training-induced thermoregulatory status. To circumvent these issues, future studies may want to standardize physical activity prior to HA when investigating the influence of exercise-induced partial heat adaptation on HA induction (e.g., as in Ravanelli et al. 2018).

### General considerations

It should be noted that the explorative nature of the current study made it impossible to disentangle body dimensions and fitness status characteristics from sex. Although overlap existed, the females in the current study generally had a smaller body mass, higher BSA-to-mass ratio, higher body fat percentage, and a lower $\dot{{V}}$O_2peak,kg_ than the males. However, the independent effect of sex on thermoregulation may be limited. Recent research suggests that sex differences in vasomotor and sudomotor activity during compensable heat stress can mainly be explained by divergence in BSA-to-mass ratio (Notley et al. 2017). Moreover, we did not control for menstrual cycle phase, which may have increased the variability in our data (Lei et al. 2019). Menstrual cycle phases during the HSTs were randomly distributed over our female participants, so it is not likely that the thermoregulatory fluctuations associated with the menstrual cycle introduced a systematic bias.

Seeking understanding of individual responses to an intervention is rather complex. Ideally, one should implement a control group as well as repeated interventions, to exclude sources of variability that are not related to the “true” individual’s response, such as random variation and within-subject variability (Hecksteden et al. 2015). Following, the variability observed in the present study cannot be attributed only to “true” inter-individual variation in HA responsiveness. In addition, various statistical analysis techniques can be employed to support data interpretation. We aimed to determine a set of characteristics that differentiated participants with a high adaptive response from those with a low response. This analysis occasionally resulted in separation of individuals with a similar adaptive response into two distinctive groups. However, the overall adaptation response differed considerably among groups. Also, by introducing a medium responder group, we created a substantial distinction between low and high responders. The interested reader is referred to the electronic supplementary material (file1 and file2) for alternative analysis using simple least-squares linear regressions; similar conclusions can be drawn from this.

In the current study, adaptive responses were deduced from the pre- and post-HA HSTs, which employed an external workload of 1.5 W kg body mass^−1^. It can be argued that the higher absolute requirement for evaporation (*W*) in large individuals led to the greater WBSR observed during HST1 in high WBSR responders, and that this may have introduced a bias in our evaluation of the WBSR adaptation. However, high WBSR responders showed a greater WBSR and WBSR_BSA_ during HA as well, where no difference in power output existed between groups. This suggests that the greater WBSR in high WBSR responders seems unrelated to the delivered power output. In addition, Ravanelli et al. (2017) recommended to normalize the exercise-induced heat production to body mass when comparing Δ*T*_re_ and sweating responses in groups with distinct body dimensions during uncompensable heat stress. It should be noted, however, that we standardized external work rate, introducing variance in the normalized heat production that is related to the individual’s cycling efficiency. Although our approach did not eliminate variations in heat production (W kg^−1^), it presumably minimized systematic differences related to individual body dimensions. This supports the assumption that our study design facilitated unbiased comparisons among our groups with varying body dimensions.

As specified in Methods section, the WBSR was determined for the full HST (i.e., including GXT_HST_). Since we investigated pre- to post-HA changes (HST2-HST1), with similar procedures pre- and post-HA (average performance time improvement 53 s), we do not believe that this feature influenced the WBSR comparisons in the present study.

## Conclusion

The findings of our study indicate that body dimensions influence the pathway of adaptation following a 10-day controlled-hyperthermia HA protocol in warm humid conditions. Participants with a high sudomotor adaptation generally had large body dimensions (large body mass and BSA, low BSA-to-mass ratio), while participants with a high end-exercise heart rate adaptation were typically small (small body mass and BSA). Body dimensions did not vary among different levels of resting *T*_re_ and Δ*T*_re_ responsiveness. Medium and high resting *T*_re_ responders tended to have a higher baseline fitness level than low responders, but no statistically significant differences were observed. Our novel findings shed new light on the individual adaptive responses observed after HA, by identifying the individual body dimensions as an influential factor. We encourage future research into the isolated effect of body dimensions on HA induction to expand upon our observations.

## Supplementary Information

Below is the link to the electronic supplementary material.

Supplementary file1 (PDF 676 KB)

Supplementary file2 (PDF 45 KB)

## Abbreviations

BSA: Body surface area
HA: Heat acclimation
HR: Heart rate
HST: Heat stress test
*T*_re_: Rectal temperature
Δ*T*_re_: Exercise-induced rise in *T*_re_
*T*_sk_: Mean skin temperature
$\dot{{V}}$O_2peak,kg_: Maximum oxygen uptake in mL kg^−^^1^ min^−^^1^
WBSR: Whole-body sweat rate
GXT_HST_: Graded exercise test during heat stress test
