# Supplementary material for: Individual characteristics associated with the magnitude of heat acclimation adaptations
Source: Eur J Appl Physiol. 2021 Mar 1;121(6):1593–606. doi: 10.1007/s00421-021-04626-3 (PMC8144163; doi:10.1007/s00421-021-04626-3)
Supplement: Supplementary file 1 — Supplementary file1 (PDF 676 KB) [file 421_2021_4626_MOESM1_ESM.pdf]

APPENDIX

Table 4 Physiological responses of low-, medium- and high resting T_{re} responders (mean ± SD or median (Q1, Q3)) with P-values from analysis of variance (AOV) and post-hoc pairwise tests (where appropriate).

	Resting T_{re} responders						
	**Low**	**Medium**	**High**	***P* AOV**	***P* low-med**	***P* med-high**	***P* low-high**
Physiological responses HST1							
ΔT_{re} (°C)	0.7 (0.6, 1.0)	0.7 (0.7, 0.8)	0.6 (0.4, 0.7)	0.3^{np}	-	-	-
End-exercise T_{re} (°C)	38.3 ± 0.4	38.2 ± 0.3	38.2 ± 0.3	0.6	-	-	-
End-exercise T_{sk} (°C)	36.5 (36.2, 36.8)[6]	36.5 (36.4, 36.5)[7]	36.6 (36.1, 36.7)[6]	$>0.9^{np}$	-	-	-
WBSR ($g \cdot h^{-1}$)	711 (621, 806)	1203 (1049, 1369)	1038 (901, 1285)	$\mathbf{0.02^{np}}$	0.06^{np}	$>0.9^{np}$	0.06^{np}
$WBSR_{BSA}$ ($g \cdot h^{-1} \cdot m^{-2}$)	384 (332, 435)	651 (507, 681)	507 (488, 653)	$\mathbf{0.03^{np}}$	0.08^{np}	$>0.9^{np}$	0.06^{np}
HR (bpm)	161 ± 17	141 ± 15	144 ± 16	**0.04**	0.06	>0.9	0.1
Heat acclimation							
Average duration thermal drive phase (min)	36 ± 5	39 ± 6	38 ± 3	0.4	-	-	-
Cumulative adaptation impulse T_{re} (°C·min)	36346 ± 1980	37342 ± 2197	37227 ± 1226	0.5	-	-	-
Cumulative adaptation impulse T_{sk} (°C·min)	34066 ± 1798	35020 ± 2341	34908 ± 1173	0.5	-	-	-
Average HR (bpm)	140 ± 15	125 ± 8	131 ± 11	0.053	-	-	-
Total work done (kJ)	4880 ± 1091	5459 ± 1118	5447 ± 661	0.4	-	-	-
Average power output (W)	86 ± 17	94 ± 18	94 ± 11	0.5	-	-	-
Average power output thermal drive phase (W)	127 ± 26	141 ± 21	141 ± 19	0.4	-	-	-
Average power output ($W \cdot kg^{-1}$)	1.1 (1.1, 1.3)	1.2 (1.0, 1.3)	1.2 (1.1, 1.3)	$>0.9^{np}$	-	-	-
WBSR ($g \cdot h^{-1}$)	858 (731, 1103)	1420 (1262, 1622)	1152 (1020, 1407)	$\mathbf{0.04^{np}}$	0.08^{np}	0.4^{np}	0.4^{np}
$WBSR_{BSA}$ ($g \cdot h^{-1} \cdot m^{-2}$)	483 (390, 567)	733 (664, 770)	591 (519, 686)	$\mathbf{0.04^{np}}$	0.06^{np}	0.6^{np}	0.4^{np}

SD, standard deviation; Q1, first quartile; Q3, third quartile; HST, heat stress test; T_{re}, rectal temperature; ΔT_{re}, exercise-induced rise in rectal temperature; T_{sk}, mean skin temperature; HR, heart rate; End-exercise, average over last 5 min of exercise; WBSR, whole-body sweat rate; BSA, body surface area; ^{np}From non-parametric test; $^{\#}$Number of participants if less than default n = 8.

Appendix

Table 5 Physiological responses of low-, medium- and high ΔT_{re} responders (mean ± SD or median (Q1, Q3)) with P-values from analysis of variance (AOV) and post-hoc pairwise tests (where appropriate).

	**ΔT_{re} responders**						
	**Low**	**Medium**	**High**	***P* AOV**	***P* low-med**	***P* med-high**	***P* low-high**
Physiological responses HST1							
ΔT_{re} (°C)	0.7 ± 0.3	0.6 ± 0.2	1.0 ± 0.3	**0.04**	>0.9	**0.047**	0.1
End-exercise T_{re} (°C)	38.3 ± 0.3	38.1 ± 0.3	38.4 ± 0.3	0.3	-	-	-
End-exercise T_{sk} (°C)	36.3 ± 0.4[6]	36.5 ± 0.3[7]	36.5 ± 0.3[6]	0.4	-	-	-
WBSR ($g \cdot h^{-1}$)	1183 ± 298	1120 ± 308	806 ± 326	0.053	-	-	-
$WBSR_{BSA}$ ($g \cdot h^{-1} \cdot m^{-2}$)	598 ± 117	560 ± 153	411 ± 126	**0.02**	>0.9	0.1	**0.03**
HR (bpm)	147 ± 17	145 ± 16	155 ± 20	0.5	-	-	-
Heat acclimation							
Average duration thermal drive phase (min)	38 ± 4	38 ± 5	36 ± 5	0.7	-	-	-
Cumulative adaptation impulse T_{re} (°C·min)	37238 ± 1617	37038 ± 1881	36639 ± 2136	0.8	-	-	-
Cumulative adaptation impulse T_{sk} (°C·min)	35118 (33526, 35563)	34215 (33766, 35211)	33685 (32949, 34975)	0.6[np]	-	-	-
Average HR (bpm)	126 ± 11	135 ± 11	134 ± 17	0.4	-	-	-
Total work done (kJ)	5114 ± 746	5673 ± 1172	5000 ± 948	0.4	-	-	-
Average power output (W)	88 ± 11	98 ± 18	87 ± 16	0.3	-	-	-
Average power output thermal drive phase (W)	135 ± 24	145 ± 22	128 ± 20	0.3	-	-	-
Average power output ($W \cdot kg^{-1}$)	1.1 ± 0.1	1.3 ± 0.2	1.2 ± 0.3	0.5	-	-	-
WBSR ($g \cdot h^{-1}$)	1367 ± 359	1346 ± 435	1003 ± 383	0.1	-	-	-
$WBSR_{BSA}$ ($g \cdot h^{-1} \cdot m^{-2}$)	693 ± 154	673 ± 214	512 ± 157	0.1	-	-	-

SD, standard deviation; Q1, first quartile; Q3, third quartile; HST, heat stress test; T_{re}, rectal temperature; ΔT_{re}, exercise-induced rise in rectal temperature; T_{sk}, mean skin temperature; HR, heart rate; End-exercise, average over last 5 min of exercise; WBSR, whole-body sweat rate; BSA, body surface area; [np]From non-parametric test; [#]Number of participants if less than default n = 8.

Appendix

Table 6 Physiological responses of low-, medium- and high WBSR responders (mean ± SD or median (Q1, Q3)) with P-values from analysis of variance (AOV) and post-hoc pairwise tests (where appropriate).

	WBSR responders						
	**Low**	**Medium**	**High**	***P* AOV**	***P* low-med**	***P* med-high**	***P* low-high**
Physiological responses HST1							
ΔT_{re} (°C)	0.6 (0.6, 0.7)	0.7 (0.6, 0.7)	0.9 (0.7, 1.1)	0.2^{np}	-	-	-
End-exercise T_{re} (°C)	38.3 ± 0.4	38.2 ± 0.4	38.2 ± 0.2	0.7	-	-	-
End-exercise T_{sk} (°C)	36.5 (36.4, 36.7)^{7}	36.6 (36.5, 36.6)^{6}	36.3 (36.2, 36.7)^{6}	0.7^{np}	-	-	-
WBSR ($g \cdot h^{-1}$)	782 ± 198	1039 ± 323	1287 ± 305	**0.007**	0.2	0.3	**0.005**
$WBSR_{BSA}$ ($g \cdot h^{-1} \cdot m^{-2}$)	425 ± 108	523 ± 161	621 ± 126	**0.03**	0.5	0.5	**0.02**
HR (bpm)	151 ± 21	143 ± 12	152 ± 19	0.5	-	-	-
Heat acclimation							
Average duration thermal drive phase (min)	33 (32, 36)	37 (35, 38)	40 (38, 42)	**0.04^{np}**	0.3^{np}	0.5^{np}	0.1^{np}
Cumulative adaptation impulse T_{re} (°C·min)	34976 (34919, 35926)	36334 (35962, 37318)	37983 (37211, 38925)	**0.02^{np}**	0.2^{np}	0.2^{np}	**0.04^{np}**
Cumulative adaptation impulse T_{sk} (°C·min)	33062 (32568, 33840)	34215 (33766, 34913)	35238 (34607, 36298)	**0.03^{np}**	0.2^{np}	0.5^{np}	0.06^{np}
Average HR (bpm)	134 ± 15	131 ± 10	131 ± 15	0.9	-	-	-
Total work done (kJ)	4840 ± 1003	5668 ± 1108	5279 ± 707	0.2	-	-	-
Average power output (W)	86 ± 17	98 ± 17	89 ± 11	0.3	-	-	-
Average power output thermal drive phase (W)	118 (106, 145)	148 (129, 155)	140 (135, 142)	0.2^{np}	-	-	-
Average power output ($W \cdot kg^{-1}$)	1.3 ± 0.2	1.3 ± 0.2	1.1 ± 0.1	0.07	-	-	-
WBSR ($g \cdot h^{-1}$)	916 ± 239	1276 ± 449	1524 ± 297	**0.006**	0.1	0.5	**0.005**
$WBSR_{BSA}$ ($g \cdot h^{-1} \cdot m^{-2}$)	499 ± 137	640 ± 220	738 ± 129	**0.03**	0.3	0.8	**0.03**

SD, standard deviation; Q1, first quartile; Q3, third quartile; HST, heat stress test; T_{re}, rectal temperature; ΔT_{re}, exercise-induced rise in rectal temperature; T_{sk}, mean skin temperature; HR, heart rate; End-exercise, average over last 5 min of exercise; WBSR, whole-body sweat rate; BSA, body surface area; ^{np}From non-parametric test; $^{\#}$Number of participants if less than default n = 8.

Appendix

Table 7 Physiological responses of low-, medium- and high HR responders (mean ± SD or median (Q1, Q3)) with P-values from analysis of variance (AOV) and post-hoc pairwise tests (where appropriate).

	HR responders						
	**Low**	**Medium**	**High**	***P* *AOV***	***P* *low-med***	***P* *med-high***	***P* *low-high***
Physiological responses HST1							
ΔT_{re} (°C)	0.5 (0.4, 0.7)	0.7 (0.6, 0.8)	0.8 (0.7, 1.0)	0.1^{np}	-	-	-
End-exercise T_{re} (°C)	38.1 ± 0.3	38.3 ± 0.3	38.3 ± 0.3	0.2	-	-	-
End-exercise T_{sk} (°C)	36.3 ± 0.4	36.6 ± 0.3[5]	36.5 ± 0.2[6]	0.2	-	-	-
WBSR ($g \cdot h^{-1}$)	1250 ± 332	901 ± 372	958 ± 232	0.09	-	-	-
$WBSR_{BSA}$ ($g \cdot h^{-1} \cdot m^{-2}$)	597 ± 147	457 ± 167	515 ± 121	0.2	-	-	-
HR (bpm)	138 ± 10	150 ± 16	158 ± 20	**0.048**	0.4	>0.9	**0.047**
Heat acclimation							
Average duration thermal drive phase (min)	39 (37, 41)	36 (33, 39)	34 (33, 38)	0.2^{np}	-	-	-
Cumulative adaptation impulse T_{re} (°C·min)	37551 (36412, 37892)	36608 (35263, 38008)	35575 (34932, 37362)	0.3^{np}	-	-	-
Cumulative adaptation impulse T_{sk} (°C·min)	34729 (34240, 35564)	34168 (33230, 35547)	33406 (32949, 35225)	0.3^{np}	-	-	-
Average HR (bpm)	128 ± 8	129 ± 15	139 ± 13	0.2	-	-	-
Total work done (kJ)	5887 ± 1007	4801 ± 780	5098 ± 885	0.06	-	-	-
Average power output (W)	100 ± 17	84 ± 11	89 ± 16	0.1	-	-	-
Average power output thermal drive phase (W)	151 ± 18	128 ± 20	128 ± 23	0.054	-	-	-
Average power output ($W \cdot kg^{-1}$)	1.2 ± 0.3	1.1 ± 0.1	1.3 ± 0.3	0.3	-	-	-
WBSR ($g \cdot h^{-1}$)	1515 ± 400	969 ± 342	1232 ± 336	**0.02**	**0.02**	0.5	0.4
$WBSR_{BSA}$ ($g \cdot h^{-1} \cdot m^{-2}$)	724 ± 185	494 ± 154	659 ± 163	**0.03**	**0.04**	0.2	>0.9

SD, standard deviation; Q1, first quartile; Q3, third quartile; HST, heat stress test; T_{re}, rectal temperature; ΔT_{re}, exercise-induced rise in rectal temperature; T_{sk}, mean skin temperature; HR, heart rate; End-exercise, average over last 5 min of exercise; WBSR, whole-body sweat rate; BSA, body surface area; ^{np}From non-parametric test; $^{\#}$Number of participants if less than default n = 8.

Appendix

Alternative analysis: Linear regression

Simple least-squares linear regressions were performed to determine the proportion of variance in the separate adaptation phenotypes (response variable) that is explained by an individual characteristic (predictor); adaptation = intercept + slope*predictor. Please refer to Fig. 4-7 to view individual data points and best-fit regression lines. Table 8-11 depict the model coefficients, the adjusted R^2 and p-values.

Table 8 Linear regression outcomes for the resting T_{re} adaptation (resting T_{re} adaptation = intercept + slope*predictor). Intercept and slope are displayed as estimate ± standard error.

Resting T_{re} adaptation				
**Predictor**	**Intercept**	**Slope**	**Adjusted R^2**	**P**
Age (y)	-0.03 ± 0.28	-0.005 ± 0.009	-0.03	0.6
Body mass (kg)	0.62 ± 0.47	-0.01 ± 0.006	0.08	0.1
BSA (m^2)	0.96 ± 0.76	-0.58 ± 0.39	0.05	0.2
BSA-to-mass ratio ($cm^2 \cdot kg^{-1}$)	-1.98 ± 1.04	0.007 ± 0.004	0.08	0.1
Body fat (%)	-0.72 ± 0.22	0.03 ± 0.01	**0.20**	**0.02**
$\dot{V}O_{2peak,kg}$ ($mL \cdot kg^{-1} \cdot min^{-1}$)	0.31 ± 0.39	-0.009 ± 0.008	0.02	0.2
Weekly exercise time$^{\times}$ (min)	0.009 ± 0.20	-0.0006 ± 0.0005	0.01	0.3

T_{re}, rectal temperature; BSA, body surface area; $\dot{V}O_{2peak,kg}$, maximum oxygen uptake relative to body mass; $^{\times}$swimming exercise excluded, n = 23.

Appendix

Table 9 Linear regression outcomes for the ΔT_{re} adaptation (ΔT_{re} adaptation = intercept + slope*predictor). Intercept and slope are displayed as estimate ± standard error.

ΔT_{re} adaptation				
**Predictor**	**Intercept**	**Slope**	**Adjusted R^2**	**P**
Age (y)	-0.07 ± 0.25	0.003 ± 0.008	-0.04	0.7
Body mass (kg)	-0.34 ± 0.43	0.005 ± 0.006	-0.01	0.4
BSA (m^2)	-0.25 ± 0.70	0.14 ± 0.36	-0.04	0.7
BSA-to-mass ratio ($cm^2 \cdot kg^{-1}$)	1.44 ± 0.93	-0.005 ± 0.004	0.05	0.1
Body fat (%)	0.31 ± 0.21	-0.01 ± 0.01	0.04	0.2
$\dot{V}O_{2peak,kg}$ ($mL \cdot kg^{-1} \cdot min^{-1}$)	-0.30 ± 0.35	0.006 ± 0.007	-0.01	0.4
Weekly exercise time$^{\times}$ (min)	-0.32 ± 0.16	0.001 ± 0.0005	**0.17**	**0.03**

T_{re}, rectal temperature; ΔT_{re}, exercise-induced rise in T_{re}; BSA, body surface area; $\dot{V}O_{2peak,kg}$, maximum oxygen uptake relative to body mass; $^{\times}$swimming exercise excluded, n = 23.

Table 10 Linear regression outcomes for the WBSR adaptation (WBSR adaptation = intercept + slope*predictor). Intercept and slope are displayed as estimate ± standard error.

WBSR adaptation				
**Predictor**	**Intercept**	**Slope**	**Adjusted R^2**	**P**
Age (y)	-69.7 ± 207.6	11.44 ± 6.42	0.09	0.09
Body mass (kg)	-571.0 ± 349.0	11.38 ± 4.57	**0.18**	**0.02**
BSA (m^2)	-797.6 ± 593.3	552.98 ± 300.80	0.09	0.08
BSA-to-mass ratio ($cm^2 \cdot kg^{-1}$)	2641.6 ± 728.9	-8.98 ± 2.78	**0.29**	**0.004**
Body fat (%)	551.6 ± 191.1	-12.81 ± 8.99	0.04	0.2
$\dot{V}O_{2peak,kg}$ ($mL \cdot kg^{-1} \cdot min^{-1}$)	283.2 ± 324.7	0.12 ± 6.18	-0.05	>0.9
Weekly exercise time$^{\times}$ (min)	23.1 ± 148.8	0.82 ± 0.41	0.12	0.06

WBSR, whole-body sweat rate; BSA, body surface area; $\dot{V}O_{2peak,kg}$, maximum oxygen uptake relative to body mass; $^{\times}$swimming exercise excluded, n = 23.

Appendix

Table 11 Linear regression outcomes for the HR adaptation (HR adaptation = intercept + slope*predictor). Intercept and slope are displayed as estimate ± standard error.

HR adaptation				
**Predictor**	**Intercept**	**Slope**	**Adjusted R^2**	**P**
Age (y)	-9.78 ± 6.35	-0.002 ± 0.20	-0.05	>0.9
Body mass (kg)	-43.70 ± 8.62	0.45 ± 0.11	**0.39**	**0.001**
BSA (m^2)	-66.80 ± 13.53	28.97 ± 6.86	**0.42**	**<0.001**
BSA-to-mass ratio ($cm^2 \cdot kg^{-1}$)	52.09 ± 21.56	-0.24 ± 0.08	**0.24**	**0.009**
Body fat (%)	-3.74 ± 5.54	-0.30 ± 0.26	0.01	0.3
$\dot{V}O_{2peak,kg}$ ($mL \cdot kg^{-1} \cdot min^{-1}$)	-16.54 ± 9.17	0.13 ± 0.17	-0.02	0.5
Weekly exercise time[×] (min)	-15.30 ± 3.86	0.02 ± 0.01	0.08	0.1

HR, heart rate; BSA, body surface area; $\dot{V}O_{2peak,kg}$, maximum oxygen uptake relative to body mass; [×]swimming exercise excluded, n = 23.

Appendix

Figure captions

Fig. 4 Resting T_{re} adaptation (response variable) against individual characteristics (predictors). Individual data points are presented, with filled points showing males and open points showing females. Black lines represent the least-squares best-fit linear regression lines. T_{re}, rectal temperature; BSA-to-mass ratio, body surface area-to-mass ratio; $\dot{V}O_{2peak,kg}$, maximum oxygen uptake relative to body mass.

Fig. 5 ΔT_{re} adaptation (response variable) against individual characteristics (predictors). Individual data points are presented, with filled points showing males and open points showing females. Black lines represent the least-squares best-fit linear regression lines. T_{re}, rectal temperature; ΔT_{re}, exercise-induced rise in T_{re}; BSA-to-mass ratio, body surface area-to-mass ratio; $\dot{V}O_{2peak,kg}$, maximum oxygen uptake relative to body mass.

Fig. 6 WBSR adaptation (response variable) against individual characteristics (predictors). Individual data points are presented, with filled points showing males and open points showing females. Black lines represent the least-squares best-fit linear regression lines. WBSR, whole-body sweat rate; BSA-to-mass ratio, body surface area-to-mass ratio; $\dot{V}O_{2peak,kg}$, maximum oxygen uptake relative to body mass.

Fig. 7 HR adaptation (response variable) against individual characteristics (predictors). Individual data points are presented, with filled points showing males and open points showing females. Black lines represent the least-squares best-fit linear regression lines. HR, heart rate; BSA-to-mass ratio, body surface area-to-mass ratio; $\dot{V}O_{2peak,kg}$, maximum oxygen uptake relative to body mass.
