# Supplementary figures and images for: Individual characteristics associated with the magnitude of heat acclimation adaptations
Source: Eur J Appl Physiol. 2021 Mar 1;121(6):1593–606. doi: 10.1007/s00421-021-04626-3 (PMC8144163; doi:10.1007/s00421-021-04626-3)

resting  $T_{re}$  adaptation ( $^{\circ}\text{C}$ )

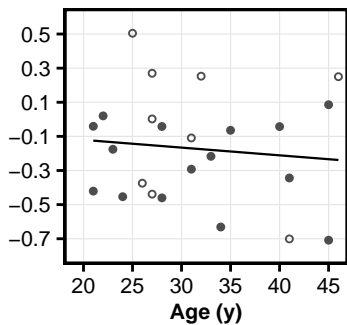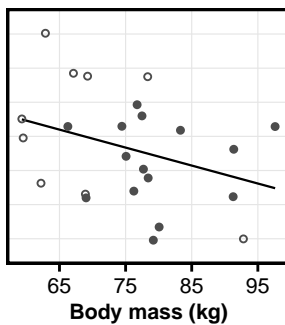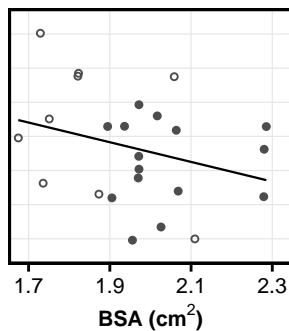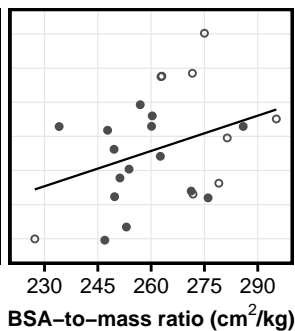

resting  $T_{re}$  adaptation ( $^{\circ}\text{C}$ )

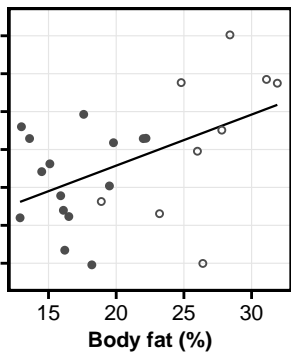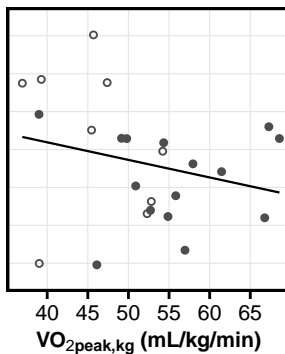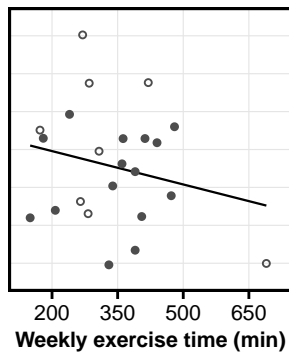

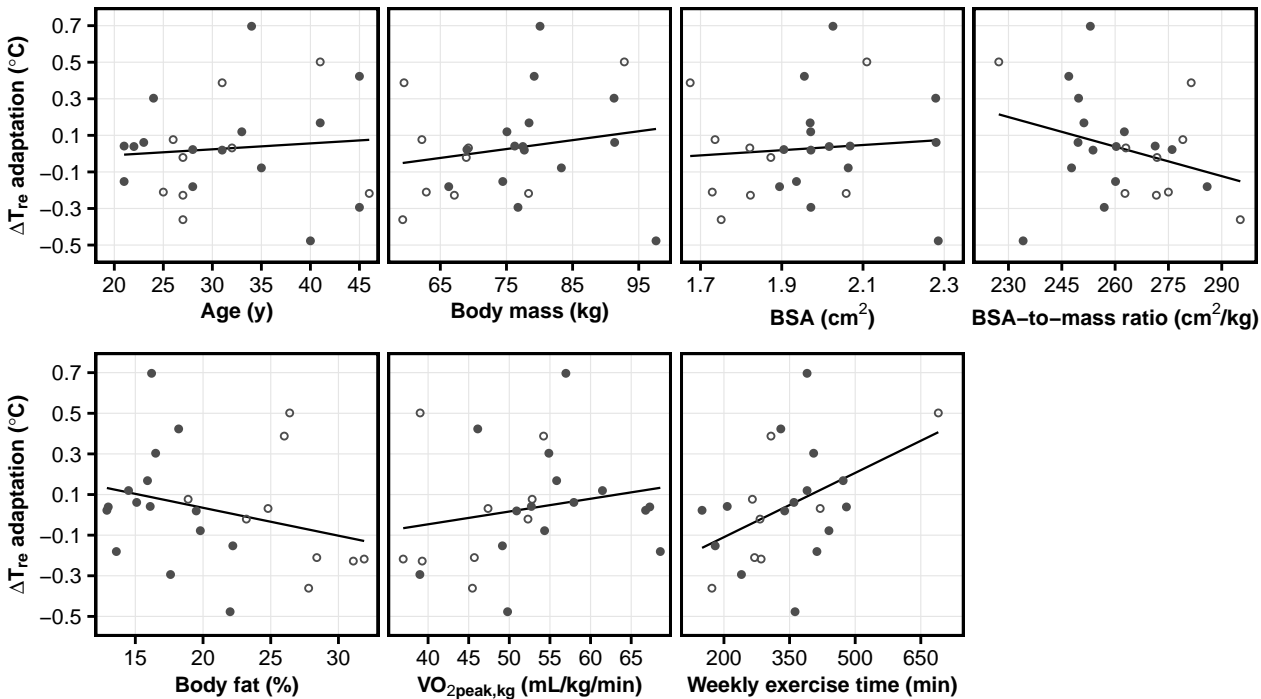

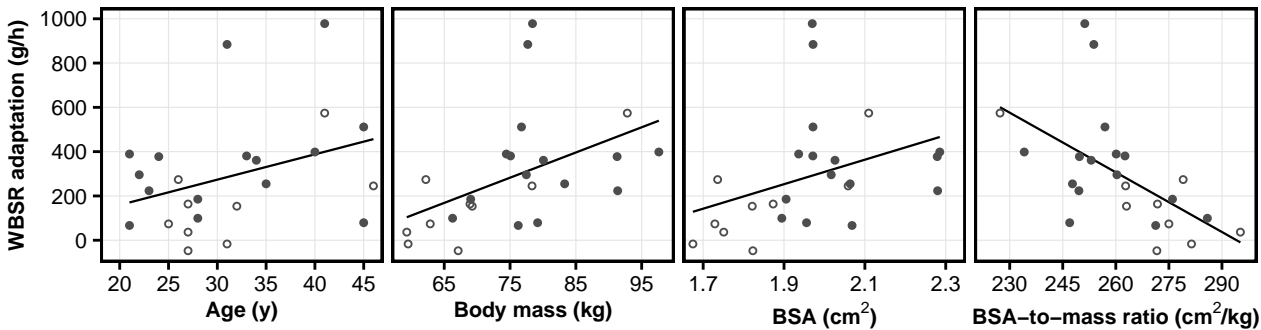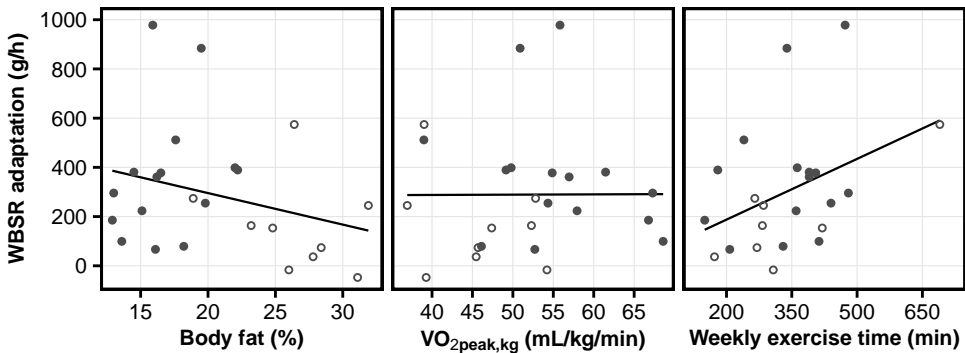

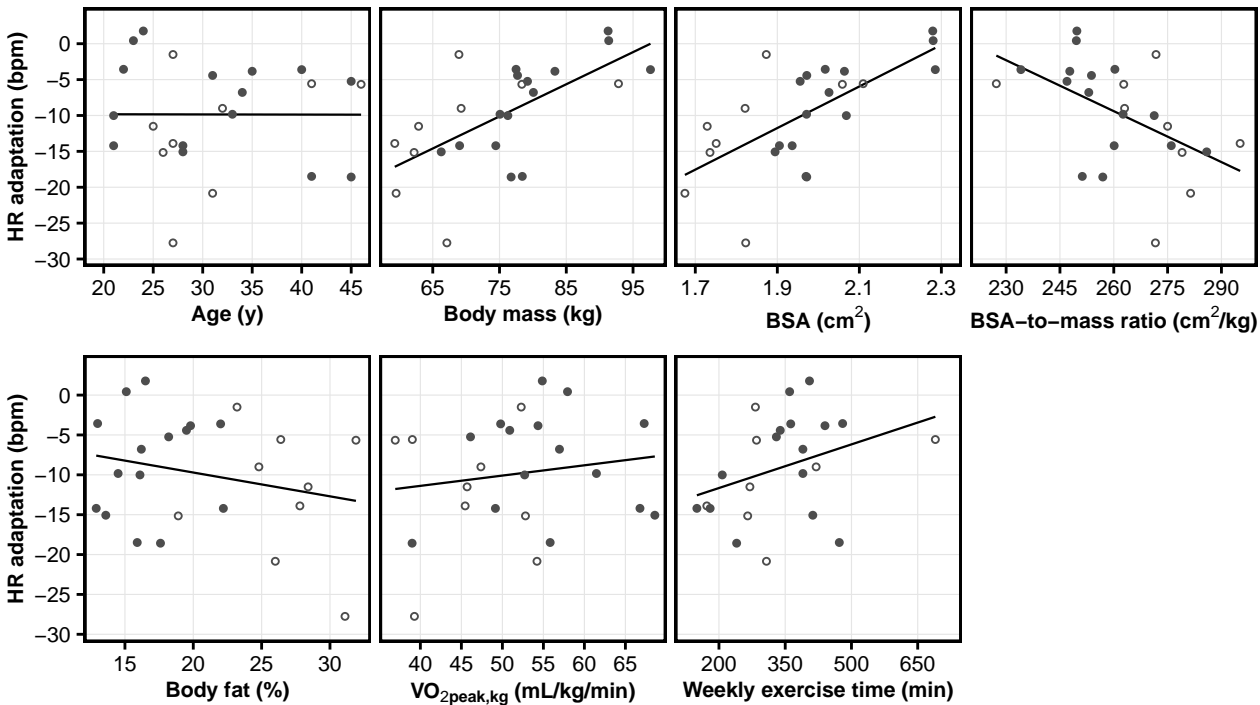

Supplement: Supplementary file 2 — Supplementary file2 (PDF 45 KB) [file 421_2021_4626_MOESM2_ESM.pdf]
